# Photoprotective Effect of Fermented and Aged Mountain-Cultivated Ginseng Sprout (*Panax ginseng*) on Ultraviolet Radiation-Induced Skin Aging in a Hairless Mouse Model

**DOI:** 10.3390/nu15071715

**Published:** 2023-03-31

**Authors:** Hee Yul Lee, Eun-Jin Kim, Du Yong Cho, Jea Gack Jung, Min Ju Kim, Jin Hwan Lee, Wanil Kim, Sang Soo Kang, Kye Man Cho, Dawon Kang

**Affiliations:** 1 Department of GreenBio Science and Agri-Food Bio Convergence Institute, Gyeongsang National University, Jinju 52727, Republic of Korea; 2 Departments of Physiology and Convergence Medical Science and Institute of Health Sciences, College of Medicine, Gyeongsang National University, Jinju 52727, Republic of Korea; 3 Department of Life Resource Industry, Dong-A University, Busan 49315, Republic of Korea; 4 Department of Biochemistry, Institute of Health Sciences, College of Medicine, Gyeongsang National University, Jinju 52727, Republic of Korea; 5 Department of Anatomy and Institute of Health Sciences, College of Medicine, Gyeongsang National University, Jinju 52727, Republic of Korea

**Keywords:** food, inner beauty, panax, skin aging, ultraviolet rays

## Abstract

Interest in foods that promote inner beauty increases with increases in exposure to ultraviolet (UV) rays and with improvements in quality of life. This study was performed to evaluate the efficacy of fermented and aged mountain-cultivated ginseng sprouts (FAMCGSs), which have higher anti-inflammatory and antioxidant effects compared to mountain-cultivated ginseng sprouts (MCGSs), as an inner beauty enhancing food. The effect of orally administered FAMCGSs on UV type B (UVB) radiation-induced skin aging was investigated in a hairless mouse model through analyzing skin parameters including epidermal thickness, transepidermal water loss (TEWL), roughness, moisture, elasticity, and collagen contents. The mice exposed to UVB had markedly greater epidermal thickness, TEWL, and skin roughness than those of the normal control (NC) group. In addition, the levels of collagen, skin moisture, and dermal elasticity were lower in the UVB radiation group than the NC group. These UVB-induced skin aging parameters were significantly lower in the groups administered FAMCGSs than in the groups not administered FAMCGSs (*p* < 0.05). These results show that FAMCGSs exhibit a photoprotective effect in mice exposed to UVB and suggest that FAMCGSs can be used as a food that promotes inner beauty and protects skin from UVB-induced photoaging.

## 1. Introduction

Environmental pollution caused by industrial development has changed the composition and function of the stratosphere. The amount of ultraviolet type B (UVB) rays reaching the earth is greater than in the past, and the occurrence of skin diseases is rising worldwide due to ozone depletion in the stratosphere [1]. The skin is the body’s outermost tissue and serves as a protective barrier between the body and the external environment. The skin has a variety of physiological functions, including blocking UV rays, preventing the intrusion of risk factors for disease such as bacteria and toxins, controlling body temperature, and maintaining body moisture [2]. The skin also serves as a medium to express health and beauty [3]. Failure to maintain healthy skin causes changes in appearance even if there is no disease, causing further stress. Since keeping our skin young and healthy is enjoyable both physically and mentally, it energizes our health overall [4]. Therefore, skincare is regarded as an essential health care task for people living in modern times. Recently, as quality of life has increased, interest in foods promoting inner beauty has also increased. The term inner beauty food refers to foods that promote inner beauty. This concept means cultivating health and beauty through food intake rather than obtaining cosmetic effects through temporary or artificial methods such as applying cosmetics [5,6].

Ginseng (*Panax ginseng* Meyer) is a perennial plant in the Panax genus of the Araliaceae family. It has long been used as a medicinal herb to prevent many ailments worldwide, particularly in Korea, China, and Japan, due to its antioxidant, anti-inflammatory, and immune-enhancing properties [7,8]. These properties originate from ginseng saponins called ginsenosides, which are the main physiologically active ingredients and quality indicators of ginseng. However, the properties and components of ginseng are highly dependent on the environment in which it is grown. It is categorized into three types based on the cultivation method and environment: cultivated ginseng (CG), mountain-cultivated ginseng (MCG), and mountain wild ginseng (MWG) [7,9]. Among these varieties, MCG has advantages over CG and MWG. Although it is grown in a mountain environment, the cultivation period is shorter than that of MWG, and the quality is superior to that of CG [7]. 

Ginseng contains major ginsenosides, including Rb1, Rb2, Re, Rc, and Rg1, which make up approximately 80% of its total content. However, their physiological activity and absorption rate in the human body are low. In contrast, minor ginsenosides, such as Rg3, Rh2, and compound K (CK), have higher physiological activity and a higher absorption rate. These minor ginsenosides (Rg3, Rh2, and CK) can be produced by aging, high temperature and pressure, and fermentation procedures [10,11]. The precursor ginsenosides (Rd and F2) for Rg3, Rh2, and CK are more abundant in ginseng leaves or sprouts than in its roots. Our recent study reported changes in the nutritional composition and biological properties of MCG sprouts (MCGSs) subjected to aging and fermentation processes [8]. The content and biological activity of ginseng metabolites and substances are changed by fermentation and aging processes as well as by the growth environment [8,12]. Microorganisms enhance the functional value of biochemical modifications, thereby increasing their phytochemical composition and content [8,13]. Antioxidant and anti-inflammatory activities were higher in fermented and aged MCGSs (FAMCGSs) than in MCGSs [8]. 

Photoaging is the result of prolonged and repeated exposed to UV radiation [14], particularly UVB radiation, which has a greater negative effect on the skin [15]. The histopathological and molecular changes that occur in photoaged skin, including DNA damage, inflammation, oxidative stress, and alteration in extracellular matrix proteins, lead to a reduction in dermal collagen, skin moisture, and elastic fibers [16]. This contributes to the aging process and can result in the appearance of wrinkles and age spots on the skin. In vitro and in vivo studies have shown that oral administration of phytochemicals with antioxidant activity, such as polyphenols, flavonoids, and vitamin C, can provide photoprotection for human skin [17,18]. FAMCGSs contain higher amounts of flavonoids and phenols than MCGSs, resulting in higher antioxidant activity [8]. FAMCGSs also have high nutritional and functional values. Therefore, FAMCGSs are expected to be highly applicable as an inner beauty food. However, the pharmacological efficacy of FAMCGSs remains unknown. This study was performed to determine the effect of orally administered FAMCGSs on UVB-induced skin aging in a hairless mouse model. 

## 2. Materials and Methods

### 2.1. Preparation of Fermented and Aged Mountain-Cultivated Ginseng Sprout (FAMCGS)

**Figure 1:**
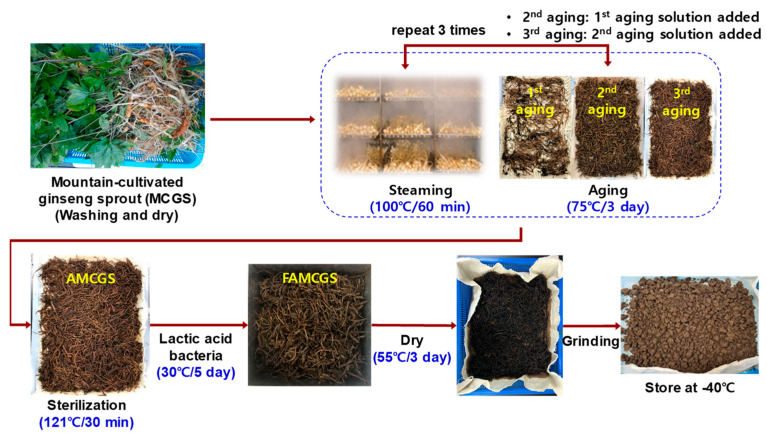
Photographs showing the production process from MCGS to FAMCGS. AMCGS, aged mountain-cultivated ginseng sprout; FAMCGS, fermented and aged mountain-cultivated ginseng sprout.

MCGSs (five years) harvested in 2017 were obtained from Jinginsengbio Farm Association Co. (Hamyang-gun, Gyeongsangnam, Republic of Korea). The procedure of FAMCGS production was performed as previously described with minor modifications [8] (Figure 1). The MCGSs were washed, steamed at 100 °C for 60 min, and then aged at 75 °C for 72 h in an aging container. There were three iterations of this procedure. The aged MCGSs were autoclaved at 121 °C for 30 min before being cooled at 30 °C. The aged MCGS sample was fermented at 30 °C for 5 days by *Lactoplantibacillus plantarum* P1201 (*L. plantarum* P1201) and *Levilactobacillus brevis* BMK484 (*L. brevis* BMK484) strains [8]. The fermented and aged MCGS samples were dried at 55 °C for 3 days. The dried FAMCGSs were ground into powders and stored at −40 °C until analysis.

To prepare FAMCGS ethanol extract concentrates for animal experiments, FAMCGS powder (20 g) was added to 400 mL of 50% ethanol. The mixture was then extracted at 40 ± 2 °C for 5 h. The extract was filtered twice through a 0.45 µm membrane filter to recover the supernatant. The supernatant was concentrated to approximately 20 °Brix using a rotary evaporator and filtered.

### 2.2. Analysis of Ginsenoside Compounds

The quantification of ginsenoside derivatives was performed using a high press liquid chromatography (HPLC) 1260 system (Agilent Technologies Inc., Waldbronn, Germany) with previously described methods [8]. The ginsenoside standards (ginsenoside Rb1, Rb2, Rb3, Rg1, Rg2, Rg3, Rd, Rd2, F1, F2, F3, F5, Rh1, Rh2, Rc, Re, Rf, Ro, compound K (CK), protopanaxdiol (PPD), and protopanaxtriol (PPT)) were purchased from KOC Biotech Co., Ltd. (Daejeon, Republic of Korea).

### 2.3. Experimental Design

An in vivo animal experiment was designed to evaluate the usability of FAMCGSs as a potential inner beauty food against UVB-induced skin aging using hairless mice, which have skin similar to humans and are susceptible to UV damage. The mice were exposed to UVB radiation for 10 weeks to mimic photoaging and skin aging parameters were then observed. To evaluate changes in the parameters, Masson’s trichrome staining was adopted to analyze epidermal thickness and dermal collagen density. In addition to histological analyses, changes in transepidermal water loss (TEWL), skin moisture content, roughness, and elasticity were also analyzed to determine whether the treatment is effective in protecting the skin barrier and dermal elasticity using each specific tool. 

### 2.4. Animal Care

The animal experiments were conducted in accordance with the recommendations of the animal care and use committee at the Korea Forestry Promotion Institute (approval number IA18-00939). SKH-1 hairless mice (female, 6 weeks old) were purchased from Orient Bio. (Animal Breeding Center, Seongnam, Republic of Korea). Ad libitum Teklad Certified Irradiated Global 18% Protein Rodent Diet (Envigo Co., Ltd., Easton, MA, USA) was provided to all mice, who were kept in a pathogen-free environment with a strict light cycle (lights on at 08:00 h and off at 20:00 h) at a temperature of 22 ± 3 °C and a relative humidity of 50 ± 20%.

### 2.5. Ultraviolet Type B (UVB)-Exposed Skin Aging Model

Seven-week-old female SKH-1 mice (*n* = 30) were assigned to six groups (*n* = 5 per each group): a no-radiation normal control group (NC), a UVB-exposed group (UV), three groups administered different concentrations of FAMGCS (UV + 150 FAMCGS, UV + 300 FAMCGS, and UV + 600 FAMCGS), and a hyaluronic acid (HA)-administered group (UV + HA). Except for the NC group, the five groups were exposed to UVB for 10 weeks using a UV-irradiation system (LK3500 HD2101.1, Lucky Scientech Co. Ltd., Bucheon, Republic of Korea) with UVB (Sankyo-Denki G6T5E UVB linear lamp, Sankyo Co., Ltd., Kanagawa, Japan). A UV radiometer was used to determine the minimal erythema dose (MED) from the UV irradiation system. MED measurements were performed with minor modification to the methods described in previous studies [19,20]. A 30 cm distance from the light source was used to determine the irradiation intensity. Erythema formation was detected after 24 h when varied UV light doses were applied to mouse dorsal skin to determine the 1 MED. The 1 MED ranged from 30 to 50 mJ/cm^2^. UVB was applied at MED three times a week to cause skin aging for a total of 10 weeks. The 1 MED, 2 MED, and 3 MED were applied in the first week, the second week, and third week of UV irradiation, respectively. The 4 MED was applied from the fourth week to the tenth week. At 10 weeks, the cervical dislocation method was used to scarify the mice. Skin tissues were rapidly separated from the mice and placed in a 4% paraformaldehyde solution or at −70 °C for further experimentation.

### 2.6. Measurement of Skin Moisture Content and Transepidermal Water Loss (TEWL)

The measurement of TEWL was performed as described in previous studies [19,20]. The moisture content and TEWL of dorsal skin were measured before UVB exposure and at 1, 3, 6, and 10 weeks after UV exposure using a Corneometer CM825 (Courage and Khazaka Electronics, Cologne, Germany) and Tewameter TM300 (Courage and Khazaka Electronics), respectively. Each measurement was performed in triplicate on all areas of the dorsal skin.

### 2.7. Measurement of Skin Elasticity and Skin Roughness

The measurement of skin elasticity and roughness was performed with minor modification to the methods described in previous studies [20,21]. Skin elasticity was measured using a Cutometer MPA 580 (Courage and Khazaka Electronics), which works by suctioning the skin into an aperture using a probe with 450 mbar negative pressure. The measurements were taken at the same time points used for TEWL assessments.

The degree of roughness of the skin epidermis was evaluated at 10 weeks by analyzing the image after photographing (Visioscan^®^ VC98, Courage & Khazaka Electronics). 

### 2.8. Epidermal Thickness and Collagen Analysis

The skin tissues collected from the mice were fixed with 4% paraformaldehyde solution overnight at 4 °C. The fixed tissues were embedded in paraffin wax, and the paraffin-embedded tissues were then sectioned into 4-µm slices. To prepare the tissue sections for staining, the paraffin wax was removed by a process called deparaffinization. The deparaffinized skin sections were stained with Masson’s trichrome to assess the collagen levels and epidermal thickness in the tissue. The stained region was photographed using a microscope, and the epidermal thickness and dermal collagen density were measured and analyzed through the images obtained using the Axio Vision SE64 (Carl Zeiss, Germany). Determination of procollagen Type I level was performed using an ELISA kit (Abcam, Waltham, MA, USA). 

### 2.9. Statistics

The data are presented as the mean ± standard deviation (SD). To determine if there were significant differences among the groups, a statistical analysis was performed using either a one-way ANOVA/Bonferroni test or Dunnett’s T3-test (SPSS 12.0 K program, Chicago, IL, USA). A p value less than 0.05 was determined to be statistically significant. 

## 3. Results

### 3.1. Content of Ginsenoside Derivatives

**Figure 2:**
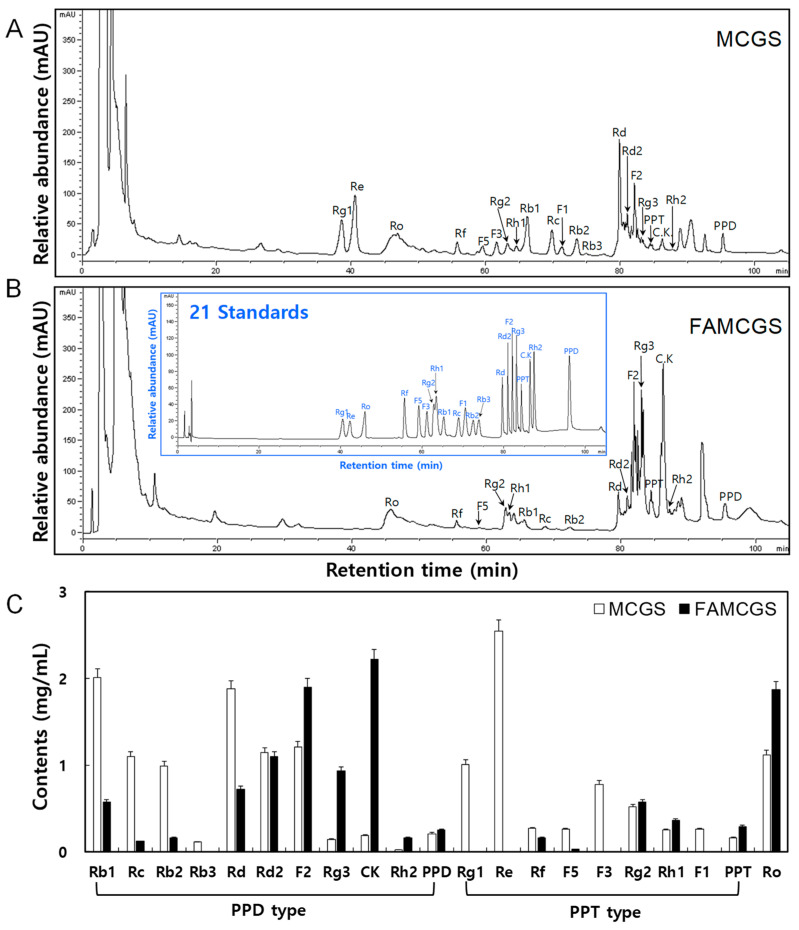
Typical HPLC chromatograms of ginsenosides in MCGSs and FAMCGSs. (**A**,**B**) Relative abundance of ginsenoside derivatives in 50% EtOH extract concentrates of MCGS (**A**) and FAMCGS (**B**). The HPLC chromatogram of the 21 standards are shown inside Figure 1B. (**C**) Comparison of ginsenoside contents in MCGSs and FAMCGSs. All values are presented as the mean ± SD of pentaplicate determinations. PPD and PPT represent protopanaxdiol and protopanaxtriol, respectively. MCGS, mountain-cultivated ginseng sprout; FAMCG, fermented and aged mountain-cultivated ginseng sprout.

The HPLC chromatograms showed the presence of 21 and 16 ginsenoside peaks in MCGSs and FAMCGSs, respectively (Figure 2A,B). In MCGSs, the ginsenosides were ranked in ascending order (Figure 2A): Re (2.54 mg/g), Rb1 (2.01 mg/g), Rd (1.88 mg/g), F2 (1.21 mg/g), Rd2 (1.14 mg/g), Ro (1.12 mg/g), Rc (1.10 mg/g), Rg1 (1.01 mg/g), Rb2 (0.99 mg/g), F3 (0.78 mg/g), Rg2 (0.52 mg/g), Rf (0.27 mg/g), F1 (0.26 mg/g), F5 (0.26 mg/g), Rh1 (0.25 mg/g), PPD (0.21 mg/g), CK (0.19 mg/g), PPT (0.16 mg/g), Rg3 (0.14 mg/g), Rb3 (0.11 mg/g), and Rh2 (0.02 mg/g). The major ginsenosides in FAMCGSs, in ascending order, were CK (2.22 mg/g), F2 (1.9 mg/g), Ro (1.87 mg/g), Rd2 (1.1 mg/g), Rg3 (0.93 mg/g), Rd (0.72 mg), Rg2 (0.57 mg/g), and Rb1 (0.57 mg/g), as shown in Figure 2B. Comparing FAMCGSs to MCGSs, there was a marked decrease in the amounts of Rb1 (2.01 → 0.57 mg/g), Rc (1.10 → 0.12 mg/g), Rb2 (0.99 → 0.16 mg/g), and Rd (1.88 → 0.72 mg/g). On the other hand, there was a notable increase in F2 (1.21 → 1.9 mg/g), Rg3 (0.14 → 0.93 mg/g), and CK (0.19 → 2.22 mg/g) in FAMCGSs (Figure 2C).

### 3.2. UVB-Exposed Skin Aging Model: Experimental Process and Body Weight Measurement

**Figure 3:**
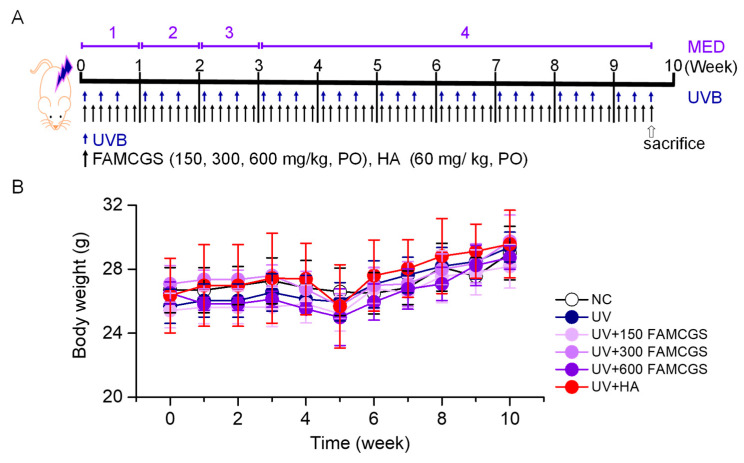
Establishment of the UV-irradiated skin aging model (**A**) A protocol for the UV-irradiated skin aging model. UVB exposure was performed for 10 weeks. FAMCGSs and HA were preadministered for 10 weeks by oral gavage. The blue and black arrows indicate exposure to UV and FAMCGSs or HA. (**B**) Changes in body weight measured during the 10 weeks of the experiment. NC, UV, FAMCGS, and HA represent normal control without UV irradiation, ultraviolet, fermented and aged mountain-cultivated ginseng sprouts, and hyaluronic acid, respectively.

Six groups were created by randomly dividing the experimental groups (*n* = 5 per each group): normal control (NC, no irradiation), UV (UV irradiation), UV + 150 FAMCGS (administration of 150 mg/kg FAMCGS before UV irradiation), UV + 300 FAMCGS (preadministration of 300 mg/kg FAMCGS), UV + 600 FAMCGS (preadministration of 600 mg/kg FAMCGS), and UV + HA (preadministration of 60 mg/kg HA). Since HA has been widely studied as a photoprotective agent, it was used as a comparative substance. The experimental protocol was summarized in Figure 3A. UV irradiation was applied to mouse dorsal skin three times per week (Monday, Wednesday, and Friday) for a total of 10 weeks. The 1 MED, 2 MED, and 3 MED were applied in the first, second, and third weeks of UV irradiation, respectively. The 4 MED was applied from the fourth to the tenth week. FAMCGSs and HA were administered by oral gavage every day (seven times per week). 

Body weight was monitored once per week for 10 weeks starting at the beginning of the experiment to assess the toxicity of FAMCGSs. There were no significant differences in body weight between the experimental groups (Figure 3B). The body weight of all groups decreased slightly at 5 weeks, but recovered again at 6 weeks and continued to increase until 10 weeks. At 10 weeks, there were no discernible variations in the experimental groups’ body weights (Figure 3B).

### 3.3. Effect of FAMCGSs on UV-Induced Skin Damage

**Figure 4:**
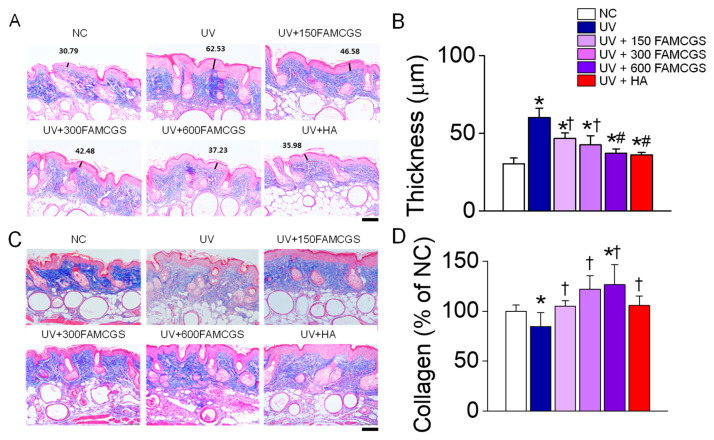
Effect of FAMCGSs on UV-induced skin histological changes. Epidermal thickness and dermal collagen density were assessed by Masson’s trichrome staining. (**A**,**B**) Changes in epidermal thickness. (**C**,**D**) Changes in dermal collagen density. Scale bar, 100 μm. Each bar represents the mean ± SD (*n* = 5 in each group). * *p* < 0.05 compared to the NC group. ^†^ *p* < 0.05 compared to the UV group. ^#^ *p* < 0.05 compared to the UV + 150 FAMCGS group.

To confirm the skin damage caused by UVB radiation, the epidermal thickness and the collagen content, which is the main component of the dermal layer, were analyzed. Epidermal hyperplasia, low density, and disordered dermal collagen fibers are histological hallmarks of photoaging skin [22]. The epidermis was dramatically more thickened in the group of mice with skin tissues exposed to UVB for 10 weeks than in the NC group (no irradiation), but the UVB-induced thickening of the epidermis was lowered in the groups pre-administered with FAMCGSs and HA (*p* < 0.05, Figure 4A). The increase in epidermal thickness caused by UVB was most significantly reduced in the group pre-administered with a high concentration of FAMCGS (UV + 600 mg/kg FAMCGS) (*p* < 0.05). The effect of 600 mg/kg FAMCGS on epidermal thickness was similar to that of HA (Figure 4B). Collagen levels were lower in the UV group than in the NC group, but the reduced collagen level was restored in the UV+FAMCGS and UV+HA groups (Figure 4C). The collagen level was considerably higher in the 300 and 600 mg/kg FAMCGS pre-administration groups than in the 150 mg/kg FAMCGS group (*p* < 0.05, Figure 3D). The collagen level was the highest in the UV + 600 mg/kg FAMCGS group. 

### 3.4. Effect of FAMCGSs on the Skin Barrier

**Figure 5:**
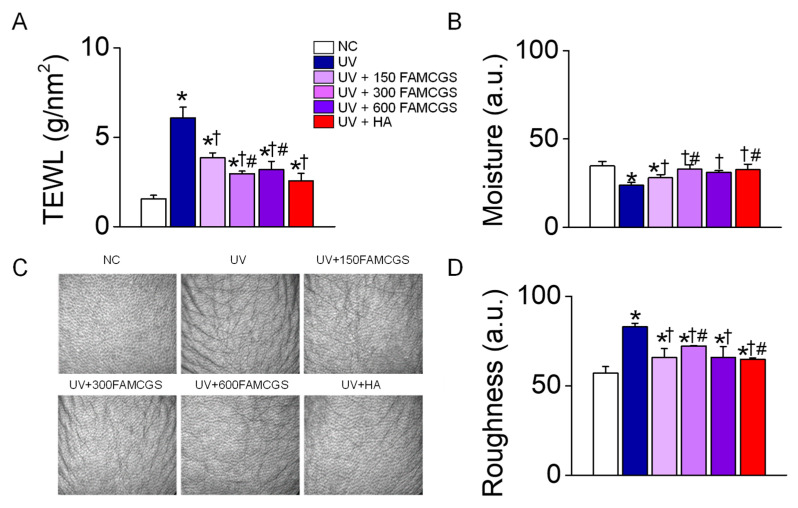
Dose-dependent effect of FAMCGSs on the skin barrier. (**A**) Changes in transepidermal water loss. (**B**) Skin moisture content. (**C**) Skin roughness. Representative photographs of dorsal skin. (**D**) Summarized effect of FAMCGSs on UV-induced skin roughness. Each bar represents the mean ± SD (*n* = 5 in each group). * *p* < 0.05 compared to NC group. ^†^ *p* < 0.05 compared to the UV group. ^#^ *p* < 0.05 compared to the UV + 150 FAMCGS group.

The changes in transepidermal water loss (TEWL), skin moisture content, and roughness were also measured to determine the effect of FAMCGSs on UVB-exposed skin damage. All experimental groups had similar values in the assessed variables at the start of the experiment. The NC group had no skin damage during the 10-week study period. The level of TEWL was significantly higher in the UV group than in the NC group (Figure 5A, *p* < 0.05), whereas the TEWL in the UV+FAMCGS and UV+HA groups was significantly lower (*p* < 0.05). The UV+300 FAMCGS and UV+600 FAMCGS groups showed a much stronger reduction than the UV+150 FAMCGS group (*p* < 0.05).

The skin moisture content showed a significant decrease in the UV group, whereas the level of recovery was significantly higher in the UV+FAMCGS and UV+HA groups than in the UV group (Figure 5B, *p* < 0.05). The skin moisture recovery was the highest in the UV + 300 FAMCGS group. 

Skin roughness is explained by epidermal wrinkles in addition to thickening of the epidermis. The roughness was higher in the UV group than in the NC group, but the roughness observed in the UV group was markedly lower in the UV+FAMCGS and UV+HA groups (Figure 5C). The roughness was significantly lower in the UV+150 FAMCGS and UV+600 FAMCGS groups than in the UV+300 FAMCGS group (Figure 5D, *p* < 0.05).

### 3.5. Effect of FAMCGSs on Dermal Elasticity

**Figure 6:**
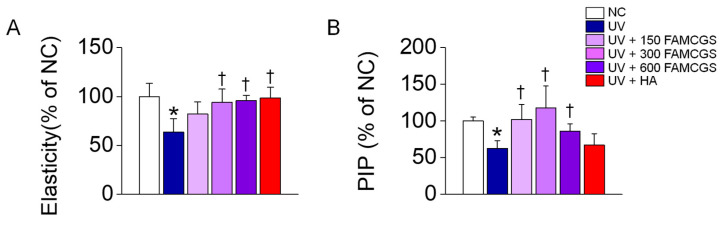
Effect of FAMCGSs on dermal elasticity. (**A**) Skin elasticity (**B**) Content of procollagen Type I C-peptide (PIP). Each bar represents the mean ± SD (*n* = 5 in each group). * *p* < 0.05 compared to the NC group. ^†^ *p* < 0.05 compared to the UV group.

Skin elasticity was lower in the UV group than in the NC group, whereas skin elasticity was significantly recovered to near the control level in the FAMCGS-administered groups (Figure 6A, *p* < 0.05). The level of procollagen Type I C-peptide (PIP) correlated with collagen levels was measured in dermal tissue lysates. PIP levels were significantly lower in the UV group than in the NC group, whereas the PIP level was recovered to the NC level in the UV+FAMCGS group (Figure 6B).

## 4. Discussion

This study reports for the first time that FAMCGSs exhibit a photoprotective effect in UVB-exposed mice. UV rays cause skin aging by destroying and reducing the production of major components that make up the epidermis and dermis [23]. In this study, mice that were exposed to UVB three times a week for 10 weeks were established as a model to mimic the changing skin condition caused by continuous UVB exposure. The skin of mice exposed to UVB continuously for 10 weeks showed an increase in epidermal thickness, TEWL, and roughness, and a decrease in collagen levels, skin moisture, elasticity, and PIP levels. However, these skin changes, which are signs of aging, were reversed in FAMCGS-administered mice. FAMCGSs are extracts of MCGSs that have been aged and fermented. FAMCGSs have higher DPPH radical scavenging activity and ferric reducing/antioxidant power than those of MCGSs and AMCGSs due to their higher content of antioxidant flavonoids and phenolic acids [8]. This powerful antioxidant activity can contribute to the prevention of skin aging caused by UVB. 

UVB-induced photoaging shows a decrease in dermal collagen, elastic fibers, and skin moisture [16]. HA is a key molecule involved in the regulation of skin moisture, and its decrease is often associated with skin aging [24]. The changes in the amount of HA under photoaging are still a matter of debate and may vary depending on several factors such as the type of experimental sample, skin area, and individual differences. However, there is much evidence that orally administered HA has an anti-photoaging effect. HA has been widely studied as a photoprotective agent [25]. Our study also found that orally administered HA had a photoprotective effect on UVB-induced skin aging parameters. The anti-photoaging effect of HA was similar to that of 600 mg/kg FAMCGS. The effect of FAMCGSs was similar to that of HA on restoring water loss and was more effective than HA in restoring collagen loss. It is inferred that FAMCGSs, which are effective in the maintaining of moisture and collagen, can greatly contribute to blocking skin aging.

Among ginsenosides, Rd2, F2, Rg3, and CK (20-O-β-D-glucopyranosyl-20(S)-protopanaxadiol) levels were higher in FAMCGSs than in MCGSs and AMCGSs. In particular, the CK concentration increased the most [8]. CK, which does not exist in natural ginseng, has been isolated by various biotransformation techniques from the major ginsenosides Rc, Rb1, and Rb2 through methods such as fermentation and from the gut microbiota [26,27,28]. The plasma concentration of CK when administered with fermented red ginseng extract was more than 10 times that achieved following administration with unfermented red ginseng extract [29]. CK, a significant deglycosylated metabolite, is identified in human tissues or blood after ingesting PPD ginsenosides [26] and red ginseng extracts [28] orally, indicating that CK is a metabolite of *Panax ginseng*. 

CK may play a more important role in the body because of its higher bioavailability and solubility compared to those of the parent ginsenosides [30]. In vitro studies have demonstrated that CK has antiaging effects on the skin, increasing HA production and decreasing COX-2 and MMP-1 production in UVB-exposed cells [31,32,33]. In vivo studies have been shown to have beneficial effects on skin barrier function. In atopic dermatitis-like and UVB-irradiated mouse models, CK was found to upregulate the expression of the serine protease inhibitor Kazal type-5 [34]. In addition, CK inhibits expression of regenerating islet-derived protein 3 gamma expression in an imiquimod-induced psoriasis mouse model [35]. These findings indicate that CK plays an important role in the defense and antiaging mechanisms of the skin. Our study indirectly suggests the antiaging effect of CK in a model of sustained UVB exposure photoaging. 

In our study, FAMCGSs (150, 300, or 600 mg/kg) administered for 10 weeks showed no toxicity in mice. FAMCGSs contain 5.05 mg/mL ginsenoside Rd2, F2, Rg3, and CK. In addition to CK, the levels of Rd2, F2, and Rg3 were increased in FAMCGSs. These molecules are PPDs. Except for Rd, the levels of Rg3, F2, and CK did not show a significant difference between red ginseng (RG) and fermented red ginseng (FRG). However, after ingestion, Rd, F2, and CK concentrations in serum are dramatically higher in the FRG-administered group than in the RG-administered group [28]. F2 is a metabolite intermediate between Rd and CK [36]. Rg3 is a characteristic component of RG [37]. Rg3 has not been studied much compared to Rb1, Rg1, and Rb2. In general, Rg3 makes up less than 0.1% of the ginsenosides in ginseng, which is a very small amount. However, similarly to Rb1 and Rg1, it has pharmacological actions such as anti-inflammatory, anticancer, anti-aging, and anti-metabolic syndrome effects [38]. Rg3, F2, and CK inhibit the activity of sodium-glucose cotransporters 1, providing a potential strategy for antihyperlycemia and antidiabetic treatments [39]. Although the composition varies depending on the processing methods, the metabolites of ginsenosides are generally the same. Unlike RG extract, FAMCGS extract contains many major ginsenoside metabolites, so it is considered to be more effective than other ginseng extracts.

Table 1 shows the anti-photoaging effect of ginseng-derived products, including FAMCGSs. Numerous studies have demonstrated that ginseng has photoprotective effects in hairless mouse models. However, due to the different experimental conditions in each study, it is challenging to accurately compare which ginseng-derived substance is the best for photoaging. Nevertheless, it is certain that ginseng ingredients are effective in preventing skin aging. Considering both functionality and economic aspects, greater added value can be obtained if sprouts with a shorter cultivation period, rather than root ginseng, are used for the ginseng-derived products. In this respect, FAMCGSs have potential as an excellent functional food. 

One downside of studying foods and their extracts is that constituent quantities might fluctuate depending on a variety of factors, such as the production process and harvest timing. However, if the indicator material or major ingredient of natural food products is discovered by ingredient analysis and function tests, these flaws can be resolved. Furthermore, it is envisaged that the use of natural products will rise if the mechanisms of action of substances are studied simultaneously. Levels of Rd2, F2, Rg3, and particularly CK rise as MCGSs age and ferment to such a degree that they can be considered as a major component of FAMCGSs. Although the mechanism of action of FAMCGSs was not investigated in this study, earlier research has investigated the mechanism of action of its single major component. The utility of FAMCGSs as an inner beauty food will rise if further studies investigate an antiaging mechanism.

## 5. Conclusions

The administration of FAMCGSs resulted in a significant reduction of UV-induced skin aging parameters compared to the NC group. Among the three concentrations of FAMCGS tested, 600 mg/kg showed the highest effect on reducing the skin aging index. Particularly, epidermal thickness, TEWL, and skin roughness were reduced by 38%, 48%, and 20% in the 600 mg/kg FAMCGS-administered group compared to the UV group, respectively. Skin moisture, collagen, and elasticity were increased by 31%, 50%, and 50% in the 600 mg/kg FAMCGS-administered group compared to the UV group, respectively. These findings suggest that FAMCGSs have a photoprotective effect in UV-exposed mice and can be used as a food to promote inner beauty and protect against photoaging caused by UVB.

## Figures and Tables

**Table 1:** Anti-aging effect of ginseng-derived products on UVB-induced photoaging in hairless mice.

Treatment	Parameters	Results	Animal	Ref.
FAMCGS	Epidermal thickness	↓	SKH-1	present study
	TEWL	↓		
	Skin roughness	↓		
	Skin moisture	↑		
	Collagen	↑		
	Elasticity	↑		
Red ginseng extract	Wrinkle	↓	SKH-1	[40]
	Collagen degradation	↓		
Ginseng leaf extract	Epidermal thickness	↓	SKH-1	[41]
	Wrinkle formation	↓		
	Collagen fiber	↑		
Red ginseng oil	Wrinkle formation	↓	HRM-2	[42]
	Epidermal thickness	↓		
	Collagen degradation	↓		
Ginseng phenolic acid extract	Epidermal thickness	↓	BALB/c	[43]
	TEWL	↓		
	Collagen degradation	↓		
Ginseng oligosaccharides	Epidermal thickness	↓	BALB/c	[44]
	TEWL	↓		
Red ginseng extracts treated with enzyme	Wrinkle formation	↓	SKH	[45]
	Epidermal thickness	↓		
	Skin dryness	↓		
Ginseng saponins, Rb1 (from red ginseng)	Epidermal thickness	↓	HR-1	[46]
	Wrinkle	↓		
	Elasticity	↑		
	Collagen fiber	↑		

## Data Availability

The study did not report any data.

## Abbreviation

AMCGS: aged mountain-cultivated ginseng sprout, CG: cultivated ginseng, CK: Compound K FAMCGS: fermented and aged mountain-cultivated ginseng sprout, FRG: fermented red ginseng HA: hyaluronic acid, HPLC: high press liquid chromatography, MCG: mountain-cultivated ginseng, MCGS: mountain-cultivated ginseng sprout, MED: minimal erythema dose, MWG: mountain wild ginseng, PIP: procollagen Type I C-peptide, PPD: protopanaxdiol, PPT: protopanaxtriol, RG: red ginseng, TEWL: transepidermal water loss, UV: ultraviolet, UVB: ultraviolet type B; UVC: ultraviolet type C.
